# The Current Use of Stem Cells in Bladder Tissue Regeneration and Bioengineering

**DOI:** 10.3390/biomedicines5010004

**Published:** 2017-01-06

**Authors:** Yvonne Y. Chan, Samantha K. Sandlin, Eric A. Kurzrock, Stephanie L. Osborn

**Affiliations:** 1 Department of Urology, Davis School of Medicine, University of California, Sacramento, CA 95817, USA; yychan@ucdavis.edu (Y.Y.C.); sksandlin@ucdavis.edu (S.K.S.); eakurzrock@ucdavis.edu (E.A.K.); 2 Stem Cell Program, Institute for Regenerative Cures, University of California, Davis Medical Center, Sacramento, CA 95817, USA

**Keywords:** urothelium, tissue engineering, bladder, stem cells

## Abstract

Many pathological processes including neurogenic bladder and malignancy necessitate bladder reconstruction, which is currently performed using intestinal tissue. The use of intestinal tissue, however, subjects patients to metabolic abnormalities, bladder stones, and other long-term sequelae, raising the need for a source of safe and reliable bladder tissue. Advancements in stem cell biology have catapulted stem cells to the center of many current tissue regeneration and bioengineering strategies. This review presents the recent advancements in the use of stem cells in bladder tissue bioengineering.

## 1. Introduction

**Figure 1:**
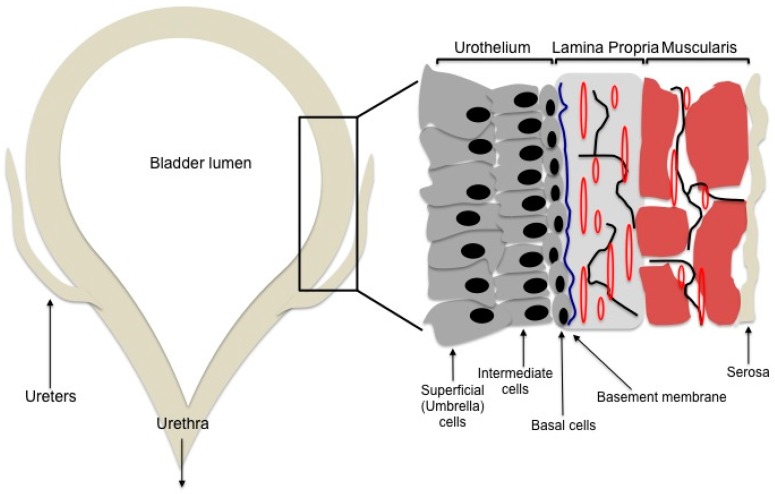
Schematic of a bladder and the different layers. The urothelium is the layer that lines the bladder lumen and forms the urine-body barrier. The lamina propria is a connective tissue layer that contains nerves and vessels (blue line = basement membrane, red lines = blood vessels, black lines = nerves). The muscularis propria is the muscular layer that provides structural support to the bladder and facilitates its physiological functions of filling and emptying. The serosa is the outermost layer.

The bladder is a reservoir that serves to store and empty urine through coordinated and complex processes. It is composed of four layers: the urothelium, lamina propria, muscularis propria, and serosa (Figure 1) [1]. The urothelium is a specialized layer that serves as a barrier between the urine and the muscle. As the primary barrier layer, the urothelium is subject to insults, such as injury, inflammation, and infection, and requires continued maintenance and repair. The urothelium is composed of three cellular layers: the basal cell layer, the intermediate layer, and the superficial layer comprised of umbrella cells. The basal layer contains urothelial stem cells and plays a critical role in urothelial regeneration. The intermediate layer rapidly regenerates urothelial tissue in cases of infection or injury. The superficial layer maintains the bladder barrier function and contains umbrella cells, which form tight junctions that limit movement of water and solutes [1,2].

Reconstruction of this intricate reservoir is necessary in many pathological processes. Patients with neurogenic bladder often require bladder augmentation for small capacity or high intravesical pressures that threaten the upper urinary tract. Patients with bladder malignancies undergo cystectomies with need for new urinary reservoirs. Bowel tissue has generally been accepted as a safe substitute for bladder tissue in these cases, but patients who have bladders reconstructed with intestinal tissue are subject to metabolic disturbances, recurrent infections, bladder stones, and increased risk for malignancy [3]. Furthermore, patients are at risk for intraoperative complications including bowel obstruction arising from the need for bowel anastomoses [4]. As such, there is increasing interest in tissue engineering to generate bladders.

Tissue engineering combines principles of cellular biology with materials science and biomedical engineering [5]. It involves fostering cellular growth on a temporary scaffold on which the cells may proliferate and generate their own extracellular matrices to replace the scaffold [6]. For years, this has been a proposed method of generating new tissues for treatment of genitourinary pathologies. The complex functions of the bladder have made this process challenging, but great advancements have been made in the last two decades that make this goal achievable.

Recent reviews have highlighted the therapeutic applications of stem cells in urological pathologies, including urinary incontinence and voiding dysfunction, as well as the use of stem cells in bladder engineering [7,8]. These reviews, however, place greater emphasis on tissue layers other than urothelium or focus on therapeutic as opposed to regenerative applications of stem cells. Herein, this review presents a comprehensive look at the recent applications of stem cells in bladder tissue bioengineering with a particular focus on the potential cell sources for urothelial regeneration and their utility in creating a bioengineered urinary reservoir. This review will also discuss the regeneration of muscle and vasculature from various stem cells and briefly cover the various scaffolds currently being studied for use in bladder bioengineering. This review summarizes the key studies that have documented in vitro and in vivo reprogramming of stem cells from various sources into urothelium. It also highlights the key papers that have reported urothelial differentiation from embryonic stem cells and induced pluripotent stem cells. As such, this review provides a special focus on urothelial regeneration and its role in bladder engineering.

## 2. Bladder Engineering

### 2.1. The Scaffold

The scaffold is a key component of tissue engineering as it guides the localization and development of the cells. Tissue generation is dependent on cellular development which is influenced by the microenvironment provided by the scaffold. As the bladder is subject to varying mechanical forces during filling and emptying, a dynamic scaffold is necessary to provide mechanical support until the engineered tissue is able to withstand these forces. Furthermore, the rate of scaffold degeneration would ideally coincide with that of tissue regeneration.

Currently, scaffolds are made of naturally derived biomaterials, acellular matrices, or synthetic polymers. Naturally derived scaffolds consist of those generated from collagen purified from human or animal tissue, chitosan, alginates, gelatin, elastin, fibrin, and silk [9,10]. Weak mechanical strength has deterred their clinical application. However, recent research suggests that silk matrices may be promising. Electrospun silk fibroin matrices were compared to bladder acellular matrices (BAM) and were found to have comparable degrees of urothelium regeneration and higher degree of vascular and smooth muscle regeneration [11]. Seth et al. also showed that silk scaffold augmented bladders were histologically and functionally similar to that of augments using small intestinal submucosa (SIS) [12]. Given silk’s biodegradability, elasticity, and mechanical strength, it may provide the structural support necessary during bladder regeneration.

Scaffolds made of acellular matrices have been widely studied. Acellular matrices are collagen rich biomaterials generated by isolating extracellular matrices from native tissues through the process of decellularization. These matrices are advantageous as they maintain the mechanical properties and structural proteins of the tissue so that engineering the biomaterial is not necessary. Common acellular matrices include small intestinal submucosa (SIS) and bladder acellular matrices (BAM). Some studies have also investigated the application of acellular amniotic membrane, acellular pericardium, and acellular dermal tissue [9]. SIS has been shown to foster bladder regeneration without ex vivo cell seeding and can be remodeled and replaced by host tissues [13,14]. BAM has similar characteristics to SIS and retains biologically active proteins necessary for tissue regeneration including vascular endothelial growth factor (VEGF) and insulin-like growth factor (IGF) [15]. Both matrices have been shown to be compatible with various urothelial and smooth muscle cell types and are used to study bladder regeneration in animal models [9].

Synthetic scaffolds are composed of polyesters such as polyglycolic acid, polylactic acid, and poly(lactic-co-glycolic acid). Compared to naturally derived grafts and acellular grafts, these matrices may be produced more efficiently with controlled properties such as degradation rate and strength [5]. Though they offer reliable mechanical properties, they often require modification with biologically active proteins inherent in acellular matrices to better regulate cellular activity. In particular, nanotechnology has been applied to improve synthetic scaffolds. Nanofibrous poly(ε-caprolactone)/poly(L-lactic acid) (PCL/PLLA) scaffolds have been shown to be biocompatible with adhesion of human urothelial and smooth muscle fiber cells [16]. Electrospun PLLA nanofiber scaffolds have also been shown to support bladder smooth muscle growth and alignment [17]. Research is ongoing regarding the application of hybrid polymers that combine the biochemical advantages offered by naturally derived scaffolds or acellular matrices and the mechanical benefits of synthetic polymers [18].

### 2.2. Urothelial Generation: Stem Cell Sources

Acellular scaffolds seeded with cells function better than non-seeded grafts. Non-seeded grafts depend on the ingrowth of the surrounding tissue which is feasible for small grafts but is challenging for larger reconstructions. Autologous cells are ideal from an immunological perspective as they circumvent issues of graft rejection. However, seeding grafts with cells from diseased bladders is not ideal as these urothelial cells are vulnerable to malignant transformation or have impaired proliferative capabilities [19,20]. Similarly, allowing ingrowth of surrounding urothelium in a diseased bladder is not prudent. With advent in stem cell technology, advances have been made in the derivation of stem cells from multiple sources (Table 1).

#### 2.2.1. Mesenchymal Stem Cells

Stem cell technology began with studies in hematopoietic stem cells found in the bone marrow, but attention has slowly shifted to mesenchymal stem cells (MSCs), a distinct population of multipotent adult stem cells. Contrary to previous theories that adult stem cells were organ and lineage specific, MSCs can differentiate into tissues of mesodermal, ectodermal, and endodermal lineages if nurtured in the proper microenvironment [46,47]. MSCs are thus an attractive source for bladder tissue engineering. Indeed, Anumanthan et al. demonstrated that MSCs differentiated into urothelium in the appropriate microenvironment. Mesenchymal cells from mouse bone marrow were combined with embryonic rat bladder mesenchymal shells. Histological evaluation revealed bladder tissue formation, and immunostaining verified uroplakin expression, confirming urothelial differentiation [48]. This was similarly shown when human bone marrow-derived mesenchymal stem cells were co-cultured with human urothelial cells and developed urothelial cell features [21]. Tian et al. further revealed that MSCs can differentiate into urothelium when only cultured in conditioned medium derived from bladder cell culture optimized for urothelial differentiation, suggesting the feasibility of cell free differentiation systems [22].

MSCs have also been found to shape the microenvironment by secreting growth factors and releasing cytokines that influence cell proliferation and angiogenesis [49]. MSCs were shown to influence cellular migration through paracrine effects in the cardiac model and is postulated to have similar roles in urological tissue regeneration [50]. MSCs are also pro-angiogenic, a property that is facilitated by expression of cysteine rich angiogenic inducer 61 (Cyr 61/CCN1). Depletion of this protein negates the angiogenic effects of MSCs [51]. Though best studied in the stroke model, MSCs may also have a role in facilitating neural regeneration [52]. Leite et al. compared integration of bladder acellular matrices in rats treated with intravenous MSCs versus control and demonstrated neuronal regeneration in the experimental group [53]. As such, MSCs may be more than a potential cell source for urothelial and smooth muscle regeneration. Further studies are needed to better elucidate the role and use of MSCs in shaping the microenvironment and in promoting angiogenesis and neuronal regeneration.

#### 2.2.2. Adult Stem Cells

In recent years, urothelium has also been derived from other sources of adult stem cells with properties resembling that of bone marrow-derived mesenchymal stem cells. Of these sources, adipose tissue is most easily accessible. Zhang et al. investigated the potential for adipose-derived stem cells (ASCs) to differentiate into urothelium. The group mixed ASCs with the immortalized human bladder urothelium cell line (SV-HUC-1) and implanted the cells into the subcutaneous tissue of athymic mice. By four weeks, approximately 70% of the ASCs expressed uroplakin Ia and 65% expressed uroplakin-II [26]. Likewise, co-culture with urothelial cells and conditioned medium induces urothelial differentiation from ASCs in vitro [23,24,25]. These studies showed that ASCs are a potential source for urothelial generation. However, the exact mechanism of differentiation remains to be fully elucidated, and their ability to function properly remains to be determined as these cells are not epithelial in origin.

Another easily accessible source of adult stem cells is urine, which harbors a subpopulation of cells with biological characteristics similar to that of mesenchymal stem cells. These cells, called “urine-derived stem cells” or USCs, have capacity for multipotent differentiation and pro-angiogenic paracrine effects [54,27]. Urine is easily obtainable without risk to the donor. A single USC may undergo 60–70 population doublings and result in large populations. Bharadwaj et al. further demonstrated that more than 90% of USCs, when cultured in appropriate conditions, expressed urothelial specific proteins including uroplakin-III and uroplakin Ia within 14 days of culture, suggesting that USCs may be a robust source of urothelium in bladder engineering [28]. While the use of autologous cells helps circumvent rejection of bioengineered tissue, most patients with bladder cancer or end stage bladders are not suitable donors. However, studies have shown that the incidence of upper tract disease in patients with known bladder tumors is low, ranging from 0.7%–3.4% [55,56]. As such, Bharadwaj et al. hypothesized that the upper tracts of patients with bladder malignancy are usually normal and demonstrated that urine collected from the upper tracts contain USCs with expansion and differentiation capabilities that make them suitable autologous alternatives for urothelium in bladder engineering [57].

A lesser studied but potential source of urothelium is hair follicle stem cells, which are capable of differentiating into cells with urothelial-like phenotype. The dermal papilla cells and cells in the adjacent region known as the dermal sheath have multipotent properties and have been directed towards adipocyte and osteocyte phenotypes [58]. Drewa et al. showed that urothelial conditioned medium increased expression of urothelial markers cytokeratin 7, 8, and 18 on hair follicle stem cells and decreased expression of hair follicle stem cell marker cytokeratin 15, suggesting that these cells may develop a urothelial-like phenotype in the appropriate environment. However, true urothelial differentiation could not be confirmed, as expression of uroplakin was not evaluated [59]. Although promising, the use of hair follicle stem cells in bladder tissue bioengineering requires more comprehensive study.

#### 2.2.3. Fetal Stem Cells

Fetal and postnatal stem cells have also been studied as potential players in bladder engineering. Human amniotic fetal stem cells can be extracted from amniotic fluid without disturbance to the embryo. They have been shown to differentiate into multiple tissue types including skin, nerve, heart, cartilage, and kidney [60,61,62,63,64]. When cultured in bladder cell conditioned medium, these cells were shown to adopt elongated and polygonal shapes typical of urothelium and to display urothelial markers including uroplakin II, cytokeratin 8, and Fibroblast growth factor 10 (FGF10) [30]. Human umbilical cord-derived mesenchymal stromal cells (HUMSCs) are a post-natal tissue source of multipotent cells. Wu et al. demonstrated that these cells are induced into urothelium when cultured in urothelial cell conditioned medium [31]. HUMSCs were seeded into bladder acellular matrix grafts for repair of bladder defects in canine models, and seeded grafts were found to be superior to unseeded grafts [32].

#### 2.2.4. Pluripotent Stem Cells

The use of mesenchymal stem cells, adult stem cells, and fetal/post-natal stem cells is limited by their poorly understood differentiation processes and the unknown long term function and safety profile of epithelial differentiation of mesenchymal lineage cells. The pluripotent nature of embryonic stem cells (ESCs) and induced pluripotent stem cells (iPSCs) therefore make them attractive candidates in cell therapy. Urothelial differentiation from pluripotent stem cells was first described in the murine model by Oottamasanthien et al., who directed mouse ESCs towards urothelial lineage through tissue recombination experiments with murine embryonic bladder mesenchyme [65]. In vitro experiments later demonstrated the significant role of retinoic acid in differentiation of murine ESCs and iPSCs to urothelium [66].

Building on the knowledge gained from mouse models, researchers have successfully induced urothelium from human ESCs and iPSCs. Our group reported a protocol detailing in vitro differentiation of urothelium from human ESCs and iPSCs using urothelial specific medium. The induction from stem cells to urothelial cells, through the definitive endoderm step, recapitulated known processes of differentiation during embryogenesis. This protocol induced 60% of the human ESCs to differentiate into urothelium, which was confirmed by uroplakin expression [33]. Kang et al. later demonstrated that human iPSCs may be induced into bladder urothelium through the definitive endoderm differentiation step using a chemically defined culture system, which would ultimately be necessary for clinical translation [34]. Moad et al. cultured iPSCs derived from urinary tract tissue in conditioned medium and produced a mixed urothelial/stromal cell culture [35]. These studies present human ESCs and iPSCs as potential sources of urothelium for future tissue engineering.

### 2.3. Muscularis Propia Generation

While the urothelium maintains the body-urine barrier through tight junctions, the muscularis propria is integral in maintaining the structural and mechanical properties of the bladder that facilitate filling and emptying. Smooth muscle cells have been derived from multiple stem cell sources (Table 1). Rodgriguez et al. cultured adipose-derived stem cells in smooth muscle differentiation medium and noted upregulated expression of smooth muscle proteins including smooth muscle cell-specific α actin, calpoin, and myosin heavy chain. These cells have also been shown to contract in response to carbachol, an effect that was blocked by atropine [36]. Smooth muscle cells have also been derived from bone marrow mesenchymal stem cells and have been shown to improve the contractility of seeded scaffolds [67,22]. Sharma et al. augmented partially cystectomized rats with poly (1,8-octaneodiol-co-citrate) elastomeric films seeded with human mesenchymal stem cells and showed that the MSCs were able to differentiate into smooth muscle cells and form more defined and organized muscular networks at 10 weeks post-transplant [37,38]. Bodin et al. seeded bacterial cellulose scaffolds with urine-derived stem cells and directed their differentiation towards urothelium and smooth muscle using specific conditioned medium, facilitating 3-dimensional growth of bladder tissues that are promising for future bladder engineering [29]. Hair follicle stem cell seeded acellular grafts also resulted in better muscle regeneration compared to that of unseeded grafts [39]. Incorporation of muscle-derived stem cells into small intestinal submucosa scaffolds also resulted in the presence of spontaneous contractile activities, which may be promising for reengineering contractile bladder augments [40]. Further studies are needed to more effectively generate cohesive muscular layers that mimic the function of a natural bladder.

### 2.4. Neovascular Generation

Recent clinical trials utilizing bioengineered bladder grafts emphasized that neovascularization of the graft is critical to prevent graft contracture and necrosis. Most research has focused on promoting angiogenesis or ingrowth of vessels into grafts using angiogenic agents such as vascular endothelial growth factor (VEGF) (Table 1). Loai et al. rehydrated bladder acellular matrices with different concentrations of VEGF and assessed angiogenesis and urothelial regeneration in mouse and porcine models. They found increased microvascular density in the grafts treated with 2 ng/g of VEGF compared to control. Similarly, grafts treated with VEGF had increased vascularization and increased urothelium and smooth muscle regeneration in the porcine model [41]. Increased microvascular density was also seen in bladder acellular matrices modified with VEGF loaded nanoparticles [42]. Incorporation of platelet-derived growth factor-BB (PDGF-BB) and vascular endothelial growth factor (VEGF) with porcine bladder acellular matrices also improved smooth muscle regeneration, vascularization, and bladder tissue contractility [43].

While promotion of angiogenesis with VEGF and similar agents may be sufficient in small bioengineered grafts, large tissues would require more extensive and rapid neovascularization that cell seeding may better facilitate. Indeed, we previously showed that graft neovascularization occurs through angiogenesis of host vessels into the proximal regions of the grafts with subsequent inoculation between host and donor vessels, which suggests that bioengineered grafts with vessels would promote early perfusion [68]. To date, there are few studies on the use of stem cells in vasculogenesis in bioengineered bladders. One recent study had shown that adipose-derived endothelial progenitor cells were able to form capillary like structures in bladder acellular matrices, suggesting these cells might serve as angiogenic cell sources in engineering bladder tissue [44]. Sharma et al. also demonstrated that co-transplantation of CD34+ hematopoietic stem/progenitor cells and MSCs resulted in improved and de novo vascularization and peripheral nerve growth in the grafts [37]. Bladder acellular matrix grafts seeded with endothelial progenitor cells modified to express VEGF exhibited enhanced neovascularization in a porcine model of partial cystectomy, suggesting that cell seeding of grafts combined with VEGF gene therapy may be the future of bladder engineering [45].

## 3. Future Directions

The ideal engineered bladder is composed of a biocompatible material that is able to sustain the mechanical forces necessary for bladder filling and emptying. The engineered urothelial layer should contain tight junctions that form an effective barrier between the bladder mesenchyme and urine. The engineered bladder should also provide environmental cues that support neovascular ingrowth and not be rejected by the host immune system. Technological advents have allowed researchers to generate reliable protocols to induce urothelial and smooth muscle cells from various sources including adult stem cells, bone marrow-derived mesenchymal stem cells, fetal/post-natal cells, embryonic stem cells, and induced pluripotent stem cells. While induced urothelial cells display urothelial markers, their ability to form watertight junctions remains to be determined. Further work is also needed to ensure proper muscle alignment and adequate neovascularization. Current research is ongoing on seeding techniques that will enable us to replicate anatomically correct and physiologically functional engineered bladders [69,70]. While great advancements have been made in stem cell engineering and urothelial differentiation, future studies will likely highlight effective seeding techniques and methods to promote angiogenesis and neural regeneration to create the ideal, dynamic urinary reservoir.

## Figures and Tables

**Table 1:** Current studies utilizing stem cells in various capacities for bladder bioengineering or regeneration.

Bladder Tissue Layer	Cell Source or Growth Factor	Model System	Major Findings	Reference(s)
Urothelium	Human bone marrow-derived mesenchymal stem cells (MSCs)	In vitro co-culture with human urothelial cells or urothelial cell conditioned medium	Induced urothelial-like cells that express cytokeratins typical of urothelium	[21]
			Exhibited epithelial characteristics via TEM	
		In vitro co-culture with human urothelium or culture in urothelial cell conditioned medium	Induced urothelium that expressed urothelial markers Uroplakin Ia (UPIa) and cytokeratins 7 and 13	[22]
	Adipose-derived stem cells (ASCs)	In vitro co-culture with human urothelial cells or urothelial cell conditioned medium	Induction of uroplakin-expressing urothelial cells in vitro	[23,24,25]
		ASCs mixed with human urothelial cell line and implanted subcutaneously into athymic mice	High expression of UPIa and Uroplakin II(UPII) at 4 weeks post-implant	[26]
	Urine-derived stem cells (USCs)	In vitro culture in urothelial specific medium and in vivo implantation of induced urothelial cells	High expression of Uroplakins in induced urothelium in vitro and in vivo	[27,28,29]
			Barrier function in vitro	
			Stratified layers of induced urothelium in vivo	
	Human amniotic fetal stem cells	In vitro co-culture with bladder cancer cell conditioned medium	Morphologically resemble urothelial cells and express UPII, cytokeratin 8 and Fibroblast growth factor 10 (FGF10)	[30]
	Human umbilical cord-derived mesenchymal stromal cells (HUMSCs)	In vitro co-culture with urothelial cell conditioned medium	Morphologically resemble urothelial cells and express UPII and cytokeratins	[31]
		HUMSCs seeded on BAMGs were used to repair bladder defects in vivo using a canine transplant model	Bladder acellular matrix grafts (BAMGs) seeded with HUMSCs had better urothelial and muscle regeneration than did non-seeded grafts	[32]
	Human embryonic stem cells (ESCs)	In vitro culture through definitive endoderm (DE) intermediary step, then induction to urothelial cells with urothelial cell-specific medium	Expression of proteins involved in urothelial fate specification during induction	[33]
			High production of urothelium determined by uroplakin expression	
	Induced pluripotent stem cells (iPSCs)	In vitro culture through DE intermediary step, then induction to urothelial cells with urothelial cell-specific medium	High production of urothelium determined by uroplakin expression	[33,34]
		Urinary tract-derived iPSCs cultured in vitro culture with urothelial cell conditioned medium	Differentiation of urothelial cells expressing UPs, cytokeratins and claudins	[35]
Muscle	Adipose-derived stem cells (ASCs)	In vitro culture in smooth muscle differentiation medium	Induced SMCs exhibited upregulation of smooth muscle proteins and contraction/relaxation properties in vitro	[36]
	Human bone marrow-derived MSCs	In vitro differentiated smooth muscle cells (via co-culture with human bladder SMCs or conditioned medium from the SMCs) were seeded onto scaffolds and transplanted in vivo	Induced smooth muscle cells increased expression of desmin in vivo and improved contractility in seeded grafts versus non-seeded grafts in vitro	[22]
		Poly (1,8-octaneodiol-co-citrate) elastomeric scaffolds were seeded with MSCs and transplanted onto cystectomized rat bladders	MSCs differentiated into SMCs within the graft and formed more organized muscular networks than did non-MSC seeded grafts	[37,38]
	Urine-derived stem cells (USCs)	USCs induced into SMCs via conditioned medium in vitro then seeded onto cellulose scaffolds and implanted subcutaneously in athymic mice	Increased SMC marker expression and functional contraction in vitro	[27,29]
			3D formation of bladder tissue in vivo	
	Hair follicle stem cells	BAMGs seeded with hair follicle stem cells in vitro then transplanted to the rat bladder	Seeded grafts showed better muscle regeneration than did non-seeded grafts	[39]
	Muscle-derived stem cells	Small intestinal submucosa (SIS) scaffolds seeded with muscle-derived stem cells were cultured in vitro	Seeded grafts exhibited spontaneous contractile activities in vitro	[40]
Blood Vessels	Vascular endothelial growth factor (VEGF)	BAMGs were hydrated with various concentrations of VEGF and utilized in a porcine model of bladder augmentation	Significant increase in vascularization, epithelialization and muscle regeneration in vivo in VEGF-hydrated BAMGs	[41]
		BAMGs seeded with VEGF-loaded nanoparticles were transplanted onto bladders of rabbits after partial cystectomy	VEGF-loaded BAMGs showed significant increase in microvessel density with decreased rate of graft contracture	[42]
	Platelet-derived growth factor-BB (PDGF-BB) + VEGF	Porcine BAMGs were loaded with Platelet derived growth factor-BB (PDGF-BB) and VEGF and transplanted into rabbits after partial cystectomy	Porcine BAMGs loaded with PDGF-BB and VEGF improved smooth muscle regeneration, vascularization and contractility	[43]
	Adipose-derived endothelial progenitor cells (ADEPCs)	ADEPCs were isolated from rat adipose tissue and cultured in vitro	ADEPCs expressed endothelial cell markers and formed capillary-like structures in BAMGs	[44]
	CD34+ hematopoietic stem/progenitor cells (HPSCs) + Bone marrow-derived MSCs	CD34+ HPSCs and MSCs were seeded onto poly (1,8-octaneodiol-co-citrate) elastomeric scaffolds and transplanted onto rat bladders after partial cystectomy	CD34+ HSPCs and MSCs increased vascularization of grafts and induced de novo vascularization and peripheral nerve growth	[37]
	VEGF-expressing endothelial progenitor cells (EPCs)	BAMGs were seeded with EPCs modified to express VEGF and used in a porcine model of partial cystectomy and transplantation	Seeded BAMGs showed enhanced vascularization versus non-EPC/VEGF seeded grafts	[45]
